# A distress thermometer with a cutoff score of ≥ 6 is the optimal point to identify highly distressed patients with advanced cancer stages in resource-limited countries without palliative care services

**DOI:** 10.3389/fonc.2023.970164

**Published:** 2023-03-15

**Authors:** Hammoda Abu-Odah, Alex Molassiotis, Justina Yat Wa Liu

**Affiliations:** ^1^ School of Nursing, The Hong Kong Polytechnic University, Hung Hom, Hong Kong, Hong Kong SAR, China; ^2^ Nursing and Health Sciences Department, University College of Applied Sciences (UCAS), Gaza, Palestine; ^3^ College of Arts, Humanities and Education, University of Derby, Derby, United Kingdom

**Keywords:** advanced cancer patients, distress thermometer, healthcare system, palliative care, screening

## Abstract

**Purpose:**

Although the distress thermometer (DT) scale has been widely validated and used in different cancer types and settings, an optimal cutoff score of DT is not defined to screen advanced cancer patients. The study aimed to define the optimal DT’s cutoff score among advanced cancer patients in resource-limited countries without palliative care services and to assess the prevalence and factors associated with psychological distress among this population.

**Methods:**

A secondary analysis was performed. Three hundred seventy-nine patients were recruited from Palestine. Participants completed the DT and the Hospital Anxiety and Depression Scale (HADS). Receiver operating characteristic analysis (ROC) was used to define the optimal cutoff score for the DT against HADS-Total ≥15. Multiple logistic regression was utilized for identifying the factors associated with psychological distress of the DT.

**Results:**

A DT cutoff score ≥ 6 correctly identified 74% of HADS distress cases and 77% of HADS non-distress cases, with a positive predictive value (PPV) and negative predictive value (NPV) of 97% and 18%, respectively. The prevalence of distress was found to be 70.7%, and the major sources of distress were related to physical (n = 373; 98.4%) and emotional problems (n = 359; 94.7%). Patients with colon (OR = 0.44, 95% CI: 0.31 – 0.62) and lymphoid cancers (OR = 0.41, 95% CI: 0.26 – 0.64) were less likely to have psychological distress than patients with other types of cancer, whereas patients with lung (OR = 1.80, 95% CI: 1.20 – 2.70) and bone cancers (OR = 1.75, 95% CI: 1.14 – 2.68) were more likely to experience it.

**Conclusion:**

A cutoff DT score of 6 appeared acceptable and effective for screening distress in patients with advanced cancer stages. Palestinian patients exhibited a high level of distress, and the high prevalence supports the argument of using a DT within the standard delivery of cancer care to identify highly distressed patients. These highly distressed patients should then be involved in a psychological intervention programme.

## Introduction

Patients with cancer experience considerable distress through their illness journeys, such as fear, coping with isolation, loss, anxiety, depression, and dependency (1). Distress is defined by the National Comprehensive Cancer Network (NCCN) as an “unpleasant emotional experience of a psychological (cognitive, behavioural, emotional), social, and/or spiritual nature that may interfere with the ability to cope effectively with cancer, its physical symptoms and its treatment” (2). Psychological distress is relatively common among cancer patients, which has been recognized as the “sixth vital sign” in cancer care (3, 4). Psychological distress is an essential outcome associated with reduced treatment compliance (5) and increased risk of health deterioration and death (6). The most common types of psychological distress patients with advanced cancer confront are anxiety and depression (7, 8). Anxiety and depression have profound negative influences on patients’ health and are associated with poor quality of life (QOL) (9) and a low level of satisfaction with medical treatments (10). Thus, identifying unrecognized cancer patients with psychological distress on time and promptly treating them is crucial in reducing the consequences of cancer side effects and enhancing their lives (11).

Several tools are available to identify psychologically distressed patients, including the Distress Thermometer (DT) (12, 13), Hospital Anxiety and Depression Scale (HADS) (14, 15), Brief Symptom Inventory-18 (BSI-18) (16) and Symptom Checklist-90 (17). Considering the length and time required to complete the previous tools, the NCCN Distress Management Panel has recommended using DT as a screening tool for distress (18). The NCCN also recommended adopting DT with a cutoff score of ≥ 4 to identify distressed cancer patients (12). Some studies adopted the recommended NCCN cutoff score for a general cancer population (19, 20). In contrast, other studies accepted a cutoff score of 3 (21, 22), a cutoff score of 5 (23, 24), or a cutoff score of 6 (25, 26). The variations in the optimal cutoff score were attributed to the cancer type (19, 20, 24, 26, 27), cultural and religious background of patients (19, 28), and clinical settings (20, 23, 29).

Despite the abundance and diversity of previous studies, most studies have been conducted in countries with high-quality cancer and palliative care (PC) services (20, 23, 29, 30). This makes it difficult to generalize the DT cutoff score in countries where PC has not been introduced in the healthcare system. No study has been conducted to define the optimal DT cutoff point in advanced cancer patients treated in a setting with no PC services introduced in the healthcare system. This study was carried out in Palestine-Gaza Strip, a country ranked by the WHO at the “capacity building activity-country” with an initiative designed to create a workforce, organizational and policy capacity for PC development (31), but no services have been integrated into their healthcare system (HCS) (32). Initiatives have been applied in PC-related areas, and most have focused on training healthcare professionals. PC services are the top priority of the Ministry of Health to be introduced into the HCS in the upcoming five years, as stated in the strategic plan for 2021–2025 (33). However, till now, PC services have not been introduced into oncological clinical practice in the Palestine-Gaza Strip. Most Gazan cancer patients’ are diagnosed in advanced stages, putting them under a high level of distress and needing psychological support. This study’s primary aim was to define the optimal cutoff score for DT among advanced cancer patients in resource-limited countries without PC services. It also aimed to find the best DT cutoff score for identifying highly distressed advanced cancer patients in stages III or IV. In addition, it identified the prevalence and factors associated with psychological distress among this population in relation to the DT data.

## Materials and methods

### Design and procedure

A secondary analysis was performed using primary data from a larger study on the unmet needs of PC patients. The study was conducted from May 2020 to August 2020 in the two hospitals in the Gaza Strip (Al-Shifa Hospital and the European Gaza Hospital) that provide cancer services for adult patients. The parent study adopted a multi-method research approach to comprehensively explore the factors and needs associated with the provision of PC services in the HCS from patients with advanced cancer, healthcare professionals and policymakers’ perspectives (34).

### Participants’ characteristics and sample size calculation

Only patients who had been diagnosed with an advanced stage (diagnosed with stages III or IV), were 18 years of age or above; were treated at one of the two hospitals that provide cancer services and who gave written consent were recruited through the convenience sampling approach. Patients with brain tumours and those exhibiting symptoms of cognitive impairment were excluded.

The patients who had appointments to visit the outpatient clinics in the hospitals were selected to participate. To identify the eligible participants, a list of patients’ names was printed from the information technology department after getting approval from the general directors of the two aforementioned hospitals. The printed list was forwarded to the heads of the oncology departments, asking them to exclude the non-eligible patients from the list. The final list of eligible patients was passed to the assigned oncology nurses who were asked to reach the selected patients and invite them to participate in the study, informing them that participation was voluntary and that they had the right to withdraw from the study at any time. Those who agreed to participate signed the informed consent form. Utilizing the sample size calculation formula described by Thompson (35), the required sample size was 368 patients. In this study, 379 patients at two hospitals in the Gaza Strip participated in the study.

### Measures

Self-administered questionnaires were adopted to collect data in this study, utilizing two instruments: the DT and HADS scales. Prior permission for their use was obtained by the original scale’s authors. Socio-demographic and medical-related variables were also collected.

#### Distress Thermometer

The Arabic version of the DT scale was used (20). It is a screening tool that has been widely used in psycho-oncologic research to identify clinically high levels of distress among cancer patients (20). The DT is a one-item measure that assesses the level of distress patients have experienced in the preceding week (36). The scale ranges from 0 (no distress) to 10 (high distress). The DT includes 36 problems answered with “*no*” or “*yes*” clustered into five domains: practical problems, family problems, emotional problems, spiritual problems, and physical problems.

#### Hospital Anxiety and Depression Scale

The Arabic version of the HADS was utilized to assess cancer patients’ anxiety and depression levels (37). It is a 14-item scale encompassing two subscales: anxiety and depression. The scores in each subscale are computed and determined to fall under one of the following three categories: normal cases (score of 0-7), borderline cases (score of 8-10), and cases (score of 11-21) (15). The cut-off point of the Arabic version of HADS for the total score was ≥ 15 and for anxiety and depression, it was ≥ 6/7. In this study, the HADS-T Arabic version had a good internal consistency with a Cronbach’s α coefficient was 0.69, with a subscale of HADS-anxiety of 0.60 and a subscale of HADS-depression of 0.62. Validation of the DT versus HADS has been adopted in many studies, showing that a total score of HADS-T ≥ 15 was the optimal cutoff score for screening distress (38, 39).

#### Socio-demographic and medical characteristics

Patient’s demographic and medical data variables were collected, such as age, gender, marital status, level of education, living conditions, cancer site, stage, type, duration since diagnosis, and current and completed treatments.

### Statistical analysis

Data were analyzed using Statistical Package for the Social Sciences (SPSS) version 25 software. Descriptive statistics were utilized to present the mean score and frequency of demographic characteristics, DT, and HADS scales. The percentages of the top 10 frequent problems/items for the distressed patients were also presented. The receiver operating characteristic (ROC) analysis was calculated to identify the optimal DT’s cutoff score against HADS-T ≥ 15. The optimal cutoff score was determined according to the point at the top left level of the curve. Sensitivity, specificity, positive predictive value (PPV), negative predictive value (NPV), and positive utility index (UI+) were calculated for each DT cutoff point against HADS-T ≥ 15. The area under the curve (AUC) of a ROC of 1 corresponded to a perfect test with 100% of sensitivity and 100% specificity was considered an optimal point to identify the DT’s cutoff score. The Youden index (*J)* was calculated to confirm the optimal cutoff DT score. Chi-square (χ^2^) analyses and t-tests were utilized as appropriate to assess for differences between the distressed and not-distressed groups with participant variables. Multivariate logistic regression analysis was performed for the purpose of identifying the factors associated with psychological distress. All statistical tests were two-tailed, and *p* values of less than 0.05 were significant.

## Results

### Participants’ characteristics

A total of 404 advanced cancer patients were approached, 25 (6.2%) refused participation, and 379 (93.8% response rate) were included in the final analysis. Participants ranged in age from 18 to 90 years old, with a mean age of 50.13 ± 14.04 years. More than half of the participants (n = 193) were male. The majority were married (n = 316; 83.4%). 50.9% of patients had stage IV cancer, and 81% received chemotherapy treatment. About 21.8% of patients had breast cancer, followed by lung cancer at15.3%. Detailed characteristics of the study participants are presented in **Table 1**.

**Table 1:** Demographic and clinical characteristics of the study subjects (N=379).

Participants’ characteristics	Total N = 379 (%)
Age in years	
Mean + SD	50.13 ± 14.04
Gender	
Male	193 (50.9)
Female	186 (49.1)
Marital status	
Married	316 (83.4)
Not married^a^	63 (16.6)
Education	
Primary and less	51 (13.5)
Secondary	243 (64.1)
University	85 (22.4)
Working status	
None	177 (46.7)
Employee	102 (26.9)
Homemaker	100 (26.4)
Monthly Income (USD) (N=359)	
Less than 250 USD	249 (69.4)
More than 250 USD	110 (30.6)
Diagnosis/type	
Breast	83 (21.8)
Colon	58 (15.3)
Lung	34 (9.0)
Bone	28 (7.4)
Prostate	20 (5.3)
Bladder	12 (3.2)
Thyroid	27 (7.1)
Lymphoid	26 (6.9)
Brain and neck	25 (6.6)
Stomach	17 (4.5)
Other	49 (12.9)
Stage	
III	186 (49.1)
IV	193 (50.9)
Current treatment	
Chemotherapy	307 (81.0)
Radiation	27 (7.1)
Surgical	16 (4.2)
Other	29 (7.7)
**DT (mean ± SD)**	6.71 ± 2.48
**HADS-T (mean ± SD)**	22.50 ± 5.52
**HADS-A (mean ± SD)**	11.35 ± 3.38
**HADS-D (mean ± SD)**	11.15 ± 3.09

SD, Standard deviation; USD, United States Dollar.

a Includes those who are single, widowed, or divorced;

b Missing data 5.3%.

The mean DT score was 6.72 ± 2.48, ranging from 0 to 10. The HADS-T score ranged from 3 to 42, with a mean score of 22.50 ± 5.52. For HADS-D, about 89.5% of patients reported signs of depression (30.9% borderline; 58.6% definitive, with a mean depression HADS score of 11.15 ± 3.09). While for the HADS-A, 87.9% of patients reported signs of anxiety (26.4% borderline; 61.5% definitive, with a mean score of 11.35 ± 3.38) (**Table 1**).

### Receiver operating characteristic analysis and the optimal cutoff score

**Figure 1:**
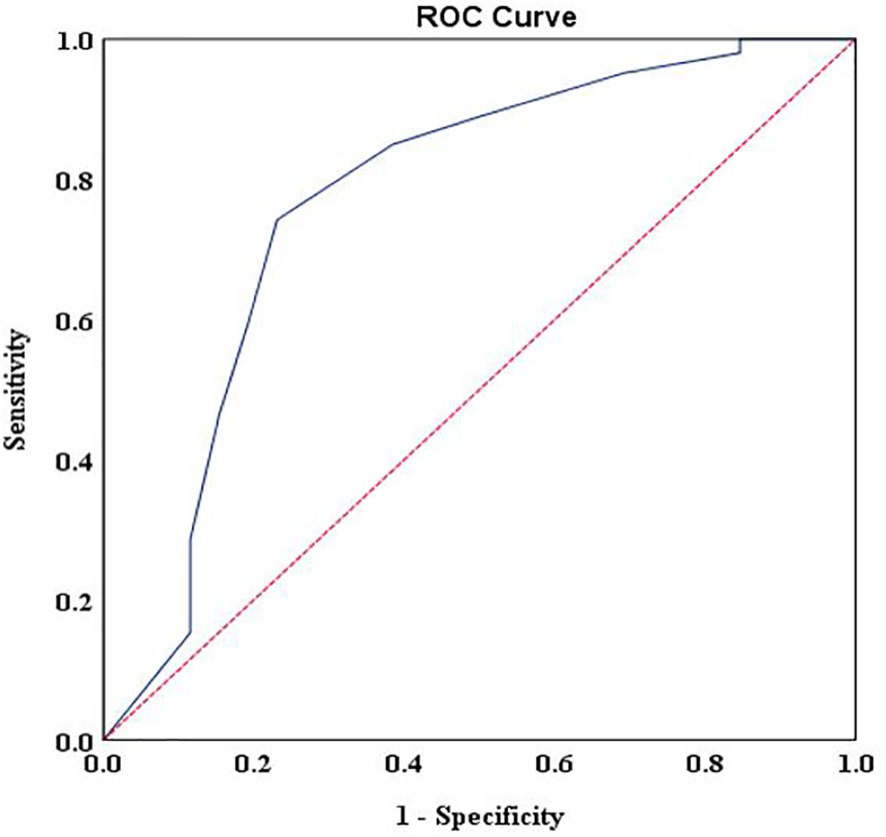
Receiver operating characteristic curve of the distress thermometer score against the Hospital Anxiety and Depression Scales-Total cutoff score > 15 for patients with advanced stages.

For patients with advanced cancer stages (stage III and IV), results showed that DT had good discriminating accuracy (AUC = 0.772, 95% CI: 0.658–0.885) between distress and no distress against HADS-T ≥15. A cutoff score of 6 on DT correctly identified 74% of HADS distress cases and 77% of HADS non-distress cases, with PPV and NPV of 97% and 18%, respectively. The *J* index and UI calculation demonstrated an accuracy of DT in screening cases (*J* = 0.51, UI+ = 0.72). Details of the accuracy of measures for DT scores according to HADS-T are presented in **Figure 1** and **Table 2**.

**Table 2:** Accuracy measures for DT scores according to HADS-T.

DT cut off score	Sensitivity	Specificity	Youden index (*J)*	Positive predictive value	Negative predictive value	Utility index +
Against HADS-T						
0/1	1.000	0.15	0.156	94.1	100	94.1
1/2	0.980	0.15	0.136	94	36.4	92.1
2/3	0.952	0.31	0.263	94.9	32	90.3
3/4	0.890	0.50	0.395	96	25	85.4
4/5	0.850	0.62	0.471	96.8	32.2	82.3
**5/6^†^**	**0.742**	**0.77**	**0.519**	**97.8**	**18.0**	**72.6**
6/7	0.595	0.81	0.411	97.7	12.8	58.1
7/8	0.465	0.85	0.319	97.6	10.4	45.3
8/9	0.286	0.89	0.180	97.1	8.4	27.8
9/10	0.153	0.89	0.156	94.7	7.1	14.5

DT, Distress Thermometer; HADS-T, Hospital Anxiety and Depression Scale-Total.

†Bold values signify the balanced cutoff point with the highest Youden index.

**Figure 2:**
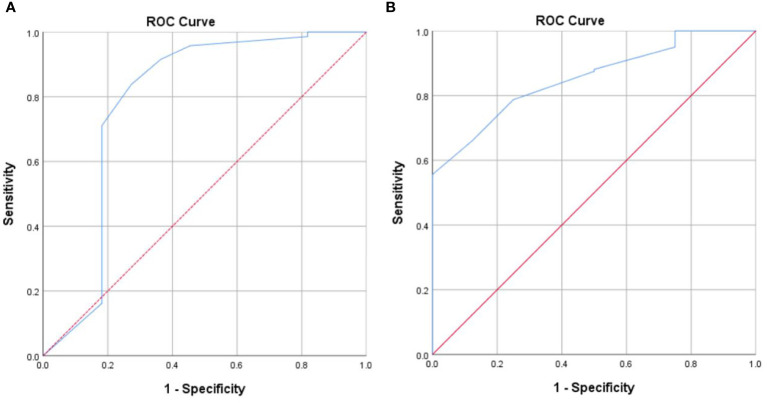
**(A)** Receiver operating characteristic curve of the distress thermometer score against the Hospital Anxiety and Depression Scales-Total cutoff score 15 for patients with stage III. **(B)** Receiver operating characteristic curve of the distress thermometer score against the Hospital Anxiety and Depression Scales-Total cutoff score ≥ 15 for patients with stage IV.

A subgroup analysis was also conducted to confirm whether a cutoff score of ≥ 6 is an appropriate point to identify highly distressed advanced cancer patients in either stage III or IV. DT had good discriminating accuracy (AUC = 0.785, 95% CI: 0.584–0.987) between distress and no distress in patients with stage III when compared to HADS-T ≥15. The cutoff score of 6 on DT also correctly identified 71.1% of HADS distress cases and 81.8% of HADS non-distress cases. The same with patients diagnosed with stage IV, the cutoff score of 6 on DT also correctly identified 78.8% of HADS distress cases and 75% of HADS non-distress cases, with good discriminating accuracy (AUC = 0.854, 95% CI: 0.757–0.950) between distress and no distress against HADS-T ≥15 (**Figures 2A, B**).

χ^2^ test of the index test results (DT ≥6) against the results of the reference standard (HADS-T ≥15) is presented in **Table 3**. The index test correctly identified 74.5% of HADS-A distress cases and 56.5% of HADS-A non-distress cases. Moreover, the index test correctly identified 73.2% of HADS-D distress cases and 51.3% of HADS-D non-distress cases. The association between index test results and HADS-A and HADS-D reached a significant level (*P*-value = < 0.000 and 0.001, respectively).

**Table 3:** Classification rates using a DT cutoff of 6 concerning HADS cases.

	Index test (DT) cutoff score ≥ 6			
Reference test (HADS) cutoff score	Above cutoff, N (%)	Below cutoff, N (%)	Chi-square OR (95% CI)	p-values
**HADS-T ≧̸ 15**			30.586 9.59 (3.73-24.64)	0.000
Above cut-off	262 (74.2)	91 (25.8)		
Below cut-off	6 (23.1)	20 (76.9)		
**HADS-A ≧̸ 8**			18.750 3.79 (2.01-7.14)	0.000
Above cut-off	248 (74.5)	85 (25.5)		
Below cut-off	20 (43.5)	26 (56.5)		
**HADS-D ≧̸ 8**			10.155 2.88 (1.47-5.64)	0.001
Above cut-off	249 (73.2)	91 (26.8)		
Below cut-off	19 (48.7)	20 (51.3)		

DT, Distress Thermometer; HADS-T, Hospital Anxiety and Depression Scale total score.

### Prevalence of distress at a cutoff score ≥ 6

**Figure 3:**
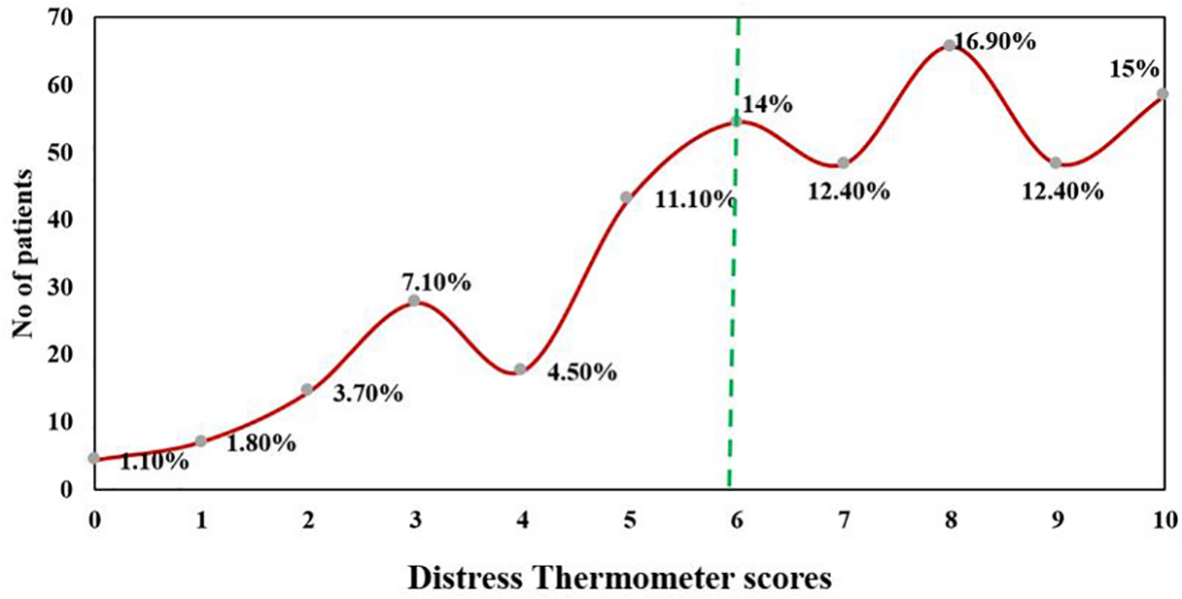
The frequency distribution of DT score under and above DT cutoff scores 6.

At DT (≥6), 70.7% of the patients (n = 268) were found to be distressed. 15% of patients reported distress at the level of 10, indicating extreme distress (**Figure 3**). The major sources of distress were related to physical (n = 373, 98.4%), emotional (n = 359, 94.7%), and practical problems (n = 324, 85.5%). Nervousness (n = 281, 73.9%), depression (n = 276, 72.8%), and fear (n = 275, 72.6%) were the main emotional sources of distress among advanced cancer patients. The top 10 frequent problems checked as a source of distress are presented in **Table 4**.

**Table 4:** Top 10 frequent problem list items checked as a source of distress among study participants.

Rank	List of problems	n (%)	Domain
1	Nervousness	281 (74.1)	Emotional
2	Depression	276 (72.8)	Emotional
3	Fears	275 (72.6)	Emotional
4	Loss of interest in activities	275 (72.6)	Emotional
5	Spiritual/religious	274 (72.3)	Spiritual
6	Sadness	265 (69.9)	Emotional
7	Fatigue	262 (69.1)	Physical
8	Worry	258 (67.5)	Emotional
9	Pain	248 (65.4)	Physical
10	Eating	246 (64.9)	Physical

### Factors influencing distress among patients with advanced cancer

All variables with a *p*-value ≤ 0.25 in univariate analysis were selected for multivariate logistic regression. Findings underscored that patients with colon (OR = 0.44, 95% CI: 0.31 – 0.62) and lymphoid cancers (OR = 0.41, 95% CI: 0.26 – 0.64) were less likely to have psychological distress than patients with other types of cancer, whereas patients with lung (OR = 1.80, 95% CI: 1.20 – 2.70) and bone cancers (OR = 1.75, 95% CI: 1.14 – 2.68) were more likely to experience it. Results also indicated that patients with stage IV (OR 1.30, 95% CI: 1.06 – 1.60) and those with emotional distress (OR 2.69, 95% CI: 1.71 – 4.23) were more likely to have psychological distress than patients with stage III and those without emotional problems (**Table 5**).

**Table 5:** Multivariate logistic regression model.

Variables	β	S.E.	Wald	OR (95% CI)	*P value*
Stage					
III	Ref.	–	–	–	–
IV	0.266	0.104	6.534	1.305 (1.06-1.60)	**0.011**
Diagnosis/type					
Breast	-0.252	0.157	2.574	0.777 (0.57-1.05)	0.109
Colon	-0.810	0.171	22.247	0.445 (0.31-0.62)	**0.000**
Lung	0.592	0.206	8.232	1.807 (1.20-2.70)	**0.004**
Bone	0.560	0.217	6.610	1.75 (1.14-2.68)	**0.010**
Prostate	10.120	0.249	0.231	0.887 (0.544-1.44)	0.631
Thyroid	-0.218	0.221	0.966	0.804 (0.52-1.24)	0.804
Lymphoid	-0.887	0.225	15.49	0.412 (0.26-0.64)	**0.000**
Brain and neck	0.323	0.229	1.97	1.38 (0.88-2.16)	0.160
Other	Ref.	–	–	–	–
Emotional problems					
No emotional problems	Ref.	–	–	–	–
Have emotional distress	0.991	0.230	18.47	2.695 (1.71-4.23)	**0.000**
Physical problems					
No physical problems	Ref.	–	–	–	–
Have physical problems	-0.991	0.230	1.073	0.653 (0.29-1.46)	0.300

HADS, Hospital Anxiety and Depression Scale. Bold values denote statistical significance at the P < 0.05 level.

## Discussion

This study was conducted to define the optimal cutoff score of DT in patients with advanced cancer stages in resource-limited countries without PC services. The study furthered the knowledge about the prevalence and risk factors associated with psychological distress among this population. A cutoff score of ≥ 6 on the DT scale is the most sensitive to be adopted for identifying advanced cancer patients with psychological distress. Patients exhibited a high level of psychological distress, anxiety, and depression. Physical and emotional related problems were the leading source of distress. The multiple logistic regression model underpinned the findings that cancer diagnosis, stage, and emotional distress were independently associated with psychological distress.

The cutoff point is crucial to dichotomize the continuous scale levels of people at risk for developing diseases and those not (40, 41). The commonly utilized methods for evaluating scale effectiveness and determining the optimal cutoff point are the AUC and the Youden index (*J*) methods (40), of which both are applied in this study to identify the cutoff point of the DT scale. The AUC is based on mapping the sensitivity by one minus specificity, where the optimal cutoff point is closed to 1 (41, 42). Our study underscored that DT at a cutoff score of 6 showed a good discriminating accuracy, which is congruent with previous literature reporting a good AUC (19, 27, 43). The Youden index (*J*) (44) is another method that based on combining sensitivity and specificity into a single measure (Sensitivity + Specificity - 1) and has a value between 0 and 1 (42). In our study, the *J* index demonstrated an accuracy of DT at a cutoff score of 6 in screening distress patients. Furthermore, the PPV and NPV were also calculated at a cutoff score of 6, resulting in fewer false-positive and false-negative rates. These are notable results that were not measured in most previous studies, based mainly on the sensitivity and specificity of the scales.

Timely identification and management of highly distressed patients are critical to enhancing their lives, which can be achieved using efficient and accurate screening tools (11). The optimal cutoff of DT is not well defined in patients with advanced cancer. There is no conclusive data regarding the optimal cutoff point because no single cutoff score has been found that increases the accuracy of DT (45). The DT with a cutoff score of ≥ 6 against HADS-T ≥ 15 is an efficacious tool for screening distress in patients with advanced cancer stages, as reported in this study. This result aligns with previous studies (25, 26). It does, however, contradicts NCCN guidelines, which recommend a cutoff score of ≥ 4 as the optimal point for screening distress (12), as well as previous studies conducted in Italy (46), Saudi Arabia (20), the United States (47) and China (48). The variations in the optimal cutoff point can be attributed to the clinical settings in which highly developed countries provided optimal care to patients, as opposed to Palestine, which has a fragmented HCS, inadequate staffing and unavailability of pain medications (32, 49), making it unable to meet the baseline needs of patients (32, 50).

Findings showed that DT ≥ 6 correctly identified 74% of advanced cancer patients as distressed and 77% as not distressed patients. Our study’s sensitivity and specificity levels are somewhat similar to a Chinese study (25), but higher than that reported in a Saudi Arabia study (20). The variations across studies are attributed to the studies’ methodological underpinnings as the former study was limited to intracranial cancer patients, while the latter study focused on all cancer stages, compared with this study that focused on cancer patients with stage III and IV cancer. Thus, a cutoff score of ≥ 6 is optimal to generalize across different cancer populations in settings with no PC services. The cutoff score of ≥ 6 will help decrease overdiagnosis due to false-positive results. Misdiagnosis of patients may burden non-distressed patients with unnecessary interventions. It may also burden and overstress the healthcare system with higher service use and costs.

Findings underscored that no associations were reported between DT and demographic and clinical variables, except for cancer diagnosis and stage. The results are in accordance with earlier studies that were also unable to find associations between DT and demographic and clinical variables (12, 51, 52). In contrast, other studies have identified an association between distress and younger patients (53), female patients and illiterate patients (54). Previous studies reported an independent association between distress and head and neck cancer, which contradicts this study that found lung and bone cancers were associated with higher distress than other cancer diagnoses (55). Psychological distress is common among patients with lung and bone cancers (56, 57). Lung cancer is the second most commonly diagnosed cancer in Palestine, comprising 11.4% of the total cancers (58). The fragile Palestinian HCS, shortage of healthcare professionals (59), and limited resources impede achieving optimal cancer services and meeting the needs of cancer patients, including lung cancer (60, 61). Furthermore, Palestinian patients with lung cancer are unfortunately diagnosed at a late stage, and they experience shortness of breath, coughing up blood, and severe chest pain (62). They are in need of oxygen therapy to alleviate their dyspnea, and prolong their survival (63, 64). However, the long-term oxygen therapy may impede their daily activities and may influence their psychological status (65).

Thus, more attention should be paid to these group of cancer patients in Palestine through psychological interventional programs to alleviate their distress.

This study reports certain limitations; adopting a cross-sectional design with non-probability sampling methods made it difficult to generalize our findings to all patients and determine the causation for any observed association. The authors determine the optimal cutoff DT score based on specific criteria, including the use of HADS; other external criteria can be used and may influence the generalizability of the findings. Despite these limitations, our findings show that determining the optimal cutoff DT score for patients with advanced cancer stages in resource-limited countries without PC services, as well as understanding the sources of distress can help healthcare professionals in identifying patients in need of urgent intervention to reduce the sources of those distresses for cancer patients. Adopting several methods for determining the optimal cutoff point is also one contribution of this study.

## Conclusion

Identifying advanced cancer patients with high distress is crucial. A cutoff DT score of 6 appeared acceptable and effective for screening of distress in this population. Palestinian patients had a high level of distress. The high prevalence supports the argument of using a DT within the standard delivery of cancer care. The highly distressed patients should then be involved in a psychological intervention programme.

## Data availability statement

The raw data supporting the conclusions of this article will be made available by the authors, without undue reservation.

## Ethics statement

Ethical approvals to undertake the study was obtained from the Human Subjects Ethics Sub-committee at The Hong Kong Polytechnic University, Hong Kong (reference number: HSEARS20200414006). Administrative approval was also obtained from the Palestinian Ministry of Health-Gaza (reference number: 476303). The patients/participants provided their written informed consent to participate in this study.

## Author contributions

HA-O, AM, and JL planned the study. HA-O and AM analyzed the data, and HA-O, AM, and JL together interpreted the findings. H-AO wrote the first draft of the manuscript and AM and JL made the final revision. All authors contributed to the article and approved the submitted version.

## References

[1] Mehnert A Hartung TJ Friedrich M Vehling S Brähler E Härter M. One in two cancer patients is significantly distressed: Prevalence and indicators of distress. Psychooncology (2018) 27(1):75–82. doi: 10.1002/pon.4464 28568377

[2] National Comprehensive Cancer Network. Distress management. Clinical practice guidelines. J Natl Compr Canc Netw (2013) 1(3):344–74. doi: 10.6004/jnccn.2003.0031 19761069

[3] Bultz BD Carlson LE. Emotional distress: the sixth vital sign in cancer care. J Clin Oncol (2005) 23(26):6440–1. doi: 10.1200/JCO.2005.02.3259 16155033

[4] Bultz BD Johansen C. Screening for distress, the 6th vital sign: where are we, and where are we going? Psycho-oncology (2011) 20(6):569–71. doi: 10.1002/pon.1986 21626609

[5] Greer JA Pirl WF Park ER Lynch TJ Temel JS. Behavioral and psychological predictors of chemotherapy adherence in patients with advanced non-small cell lung cancer. J Psychosom Res (2008) 65(6):549–52. doi: 10.1016/j.jpsychores.2008.03.005 PMC4028043 19027443

[6] Pinquart M Duberstein PR. Depression and cancer mortality: a meta-analysis. Psychol Med (2010) 40(11):1797–810. doi: 10.1017/S0033291709992285 PMC2935927 20085667

[7] Pitman A Suleman S Hyde N Hodgkiss A. Depression and anxiety in patients with cancer. BMJ (2018) 361:k1415. doi: 10.1136/bmj.k1415 29695476

[8] Lie HC Hjermstad MJ Fayers P Finset A Kaasa S Loge JH. Depression in advanced cancer – assessment challenges and associations with disease load. J Affect Disord (2015) 173:176–84. doi: 10.1016/j.jad.2014.11.006 25462414

[9] Nikbakhsh N Moudi S Abbasian S Khafri S. Prevalence of depression and anxiety among cancer patients. Caspian J Intern Med (2014) 5(3):167–70. PMC4143739 25202445

[10] Shim EJ Shin YW Jeon HJ Hahm BJ. Distress and its correlates in Korean cancer patients: pilot use of the distress thermometer and the problem list. Psychooncology (2008) 17(6):548–55. doi: 10.1002/pon.1275 17957764

[11] Carlson LE Waller A Mitchell AJ. Screening for distress and unmet needs in patients with cancer: review and recommendations. J Clin Oncol (2012) 30(11):1160–77. doi: 10.1200/JCO.2011.39.5509 22412146

[12] Holland JC Andersen B Breitbart WS Buchmann LO Compas B Deshields TL. Distress management: Clinical practice guidelines in oncology. J Natl Compr Cancer Netw (2013) 11(2):190–209. doi: 10.6004/jnccn.2013.0027 23411386

[13] Roth AJ Kornblith AB Batel-Copel L Peabody E Scher HI Holland JC. Rapid screening for psychologic distress in men with prostate carcinoma: a pilot study. Cancer (1998) 82(10):1904–8. doi: 10.1002/(SICI)1097-0142(19980515)82:10<1904::AID-CNCR13>3.0.CO;2-X 9587123

[14] Herrmann C. International experiences with the hospital anxiety and depression scale-areview of validation data and clinical results. J Psychosom Res (1997) 42(1):17–41. doi: 10.1016/S0022-3999(96)00216-4 9055211

[15] Zigmond AS Snaith RP. The hospital anxiety and depression scale. Acta Psychiatr Scand (1983) 67(6):361–70. doi: 10.1111/j.1600-0447.1983.tb09716.x 6880820

[16] Derogatis LR. BSI brief symptom inventory. In: Administration, scoring, and procedures manual. Minneapolis: National Computer Systems, (1993).

[17] Derogatis LR Lipman RS Covi L. SCL-90: an outpatient psychiatric rating scale–preliminary report. Psychopharmacol Bull (1973) 9(1):13–28. 4682398

[18] National Comprehensive Cancer Network. NCCN practice guidelines for the management of psychosocial distress. Oncology (1999) 13(5A):113–47. 10370925

[19] Donovan KA Grassi L McGinty HL Jacobsen PB. Validation of the distress thermometer worldwide: state of the science. Psycho-Oncology (2014) 23(3):241–50. doi: 10.1002/pon.3430 25160838

[20] Alosaimi FD Abdel-Aziz N Alsaleh K AlSheikh R AlSheikh R Abdel-Warith A. Validity and feasibility of the Arabic version of distress thermometer for Saudi cancer patients. PLoS One (2018) 13(11):e0207364–e. doi: 10.1371/journal.pone.0207364 PMC6241127 30427918

[21] Dolbeault S Bredart A Mignot V Hardy P Gauvain-Piquard A Mandereau L. Screening for psychological distress in two French cancer centers: feasibility and performance of the adapted distress thermometer. Palliat Support Care (2008) 6(2):107–17. doi: 10.1017/S1478951508000187 18501045

[22] Gunnarsdottir S Thorvaldsdottir GH Fridriksdottir N Bjarnason B Sigurdsson F Skulason B. The psychometric properties of the icelandic version of the distress thermometer and problem list. Psychooncology (2012) 21(7):730–6. doi: 10.1002/pon.1950 21449038

[23] Khatib J Salhi R Awad G. Distress in cancer in-patients in king Hussein cancer center (KHCC): A study using the Arabic-modified version of the distress thermometer. Psychooncology (2004) 13(1):S42–S.

[24] Bulotiene G Zalnierunaite L. Psychological distress in LITHUANIAN women with breast cancer. Rev Argent Clinica Psicologica (2011) 20(3):271.

[25] Goebel S Mehdorn HM. Measurement of psychological distress in patients with intracranial tumours: the NCCN distress thermometer. J Neurooncol (2011) 104(1):357–64. doi: 10.1007/s11060-010-0501-5 21188470

[26] Renovanz M Gutenberg A Haug M Strittmatter E Mazur J Nadji-Ohl M. Postsurgical screening for psychosocial disorders in neurooncological patients. Acta Neurochir (2013) 155(12):2255–61. doi: 10.1007/s00701-013-1884-9 24078064

[27] Ma X Zhang J Zhong W Shu C Wang F Wen J. The diagnostic role of a short screening tool–the distress thermometer: a meta-analysis. Supportive Care Cancer (2014) 22(7):1741–55. doi: 10.1007/s00520-014-2143-1 24510195

[28] Ploos van Amstel FK Tol J Sessink KH van der Graaf WTA Prins JB Ottevanger PB. A specific distress cutoff score shortly after breast cancer diagnosis. Cancer Nurs (2017) 40(3):E35–E40. doi: 10.1097/NCC.0000000000000380 27135753

[29] Ascencio-Huertas L Allende-Pérez SR Pastrana T. Associated factors of distress in patients with advanced cancer: A retrospective study. Palliat Support Care (2021) 19(4):447–56. doi: 10.1017/S1478951520001066 33222720

[30] Nguyen TQ Do TM Pham TA. Screening for psychological distress in vietnamese cancer patients: An evaluation of the distress thermometer. Cancer Med (2021) 10(21):7793–803. doi: 10.1002/cam4.4298 PMC8559463 34559957

[31] Connor SR Sepulveda Bermedo MC. Global atlas of palliative care at the end of life. United Kingdom: The World Health Organization- Worldwide Palliative Care Alliance (2014).

[32] Abu Hamad B Skaik N Abu-Odah H. Evaluation of palliative care services provided to cancer patients in the Gaza strip. JUCMS (2016) 13(2016):95–107. doi: 10.17265/1548-6648/2016.02.006

[33] Abu-Odah H Molassiotis A Liu JYW. Gathering policymakers’ perspectives as an essential step in planning and implementing palliative care services at a national level: an example from a resource-limited country. BMC Palliative Care (2022) 21(1):43. doi: 10.1186/s12904-022-00936-1 35354398 PMC8967559

[34] Abu-Odah H. An exploration of the factors and needs associated with the development of a palliative care programme into the Palestinian healthcare system from different key stakeholders’ perceptions. Hong Kong: The Hong Kong Polytechnic University (2022).

[35] Thompson S. Sampling, 3rd ed. Hoboken, New Jersey: John Wiley & Sons, Inc. (2012).

[36] Gessler S Low J Daniells E Williams R Brough V Tookman A. Screening for distress in cancer patients: is the distress thermometer a valid measure in the UK and does it measure change over time? a prospective validation study. Psychooncology (2008) 17(6):538–47. doi: 10.1002/pon.1273 17973237

[37] Terkawi AS Tsang S AlKahtani GJ Al-Mousa SH Al Musaed S AlZoraigi US. Development and validation of Arabic version of the hospital anxiety and depression scale. Saudi J anaesthesia (2017) 11(Suppl 1):S11–s8. doi: 10.4103/sja.SJA_43_17 PMC5463562 28616000

[38] Wang Y Zou L Jiang M Wei Y Jiang Y. Measurement of distress in Chinese inpatients with lymphoma. Psychooncology (2013) 22(7):1581–6. doi: 10.1002/pon.3170 22936310

[39] Vodermaier A Millman RD. Accuracy of the hospital anxiety and depression scale as a screening tool in cancer patients: a systematic review and meta-analysis. Support Care Cancer (2011) 19(12):1899–908. doi: 10.1007/s00520-011-1251-4 21898134

[40] Perkins NJ Schisterman EF. The inconsistency of "optimal" cutpoints obtained using two criteria based on the receiver operating characteristic curve. Am J Epidemiol (2006) 163(7):670–5. doi: 10.1093/aje/kwj063 PMC1444894 16410346

[41] Greiner M Pfeiffer D Smith RD. Principles and practical application of the receiver-operating characteristic analysis for diagnostic tests. Prev Veterinary Med (2000) 45(1):23–41. doi: 10.1016/S0167-5877(00)00115-X 10802332

[42] Swets JA. Measuring the accuracy of diagnostic systems. Science (1988) 240(4857):1285–93. doi: 10.1126/science.3287615 3287615

[43] Zheng B Du P Yi T Liu J Zeng Z Luo D. Effects of two translated phrases of distress thermometer on screening distress in Chinese cancer patients: A comparative study. J Clin Nurs (2019) 28(5-6):828–35. doi: 10.1111/jocn.14678 30230077

[44] Youden WJ. Index for rating diagnostic tests. Cancer (1950) 3(1):32–5. doi: 10.1002/1097-0142(1950)3:1<32::AID-CNCR2820030106>3.0.CO;2-3 15405679

[45] Hoffman BM Zevon MA D'Arrigo MC Cecchini TB. Screening for distress in cancer patients: the NCCN rapid-screening measure. Psychooncology (2004) 13(11):792–9. doi: 10.1002/pon.796 15386639

[46] Grassi L Johansen C Annunziata MA Capovilla E Costantini A Gritti P. Screening for distress in cancer patients: a multicenter, nationwide study in Italy. Cancer (2013) 119(9):1714–21. doi: 10.1002/cncr.27902 23423789

[47] Jacobsen PB Donovan KA Trask PC Fleishman SB Zabora J Baker F. Screening for psychologic distress in ambulatory cancer patients. Cancer (2005) 103(7):1494–502. doi: 10.1002/cncr.20940 15726544

[48] Deng Y-T Zhong W-N Jiang Y. Measurement of distress and its alteration during treatment in patients with nasopharyngeal carcinoma. Head Neck (2014) 36(8):1077–86. doi: 10.1002/hed.23412 23804505

[49] Abu-Odah H Molassiotis A Liu J. Challenges on the provision of palliative care for patients with cancer in low- and middle-income countries: a systematic review of reviews. BMC Palliat Care (2020) 19(1):55. doi: 10.1186/s12904-020-00558-5 32321487 PMC7178566

[50] ElMokhallalati Y Alaloul E Shatat M Shneewra T El Massri S Shaer O. The symptom burden and quality of life in cancer patients in the Gaza strip, Palestine: A cross-sectional study. PLoS One (2022) 17(1):e0262512. doi: 10.1371/journal.pone.0262512 35025966 PMC8758072

[51] Akizuki N Akechi T Nakanishi T Yoshikawa E Okamura M Nakano T. Development of a brief screening interview for adjustment disorders and major depression in patients with cancer. Cancer (2003) 97(10):2605–13. doi: 10.1002/cncr.11358 12733160

[52] Herschbach P Keller M Knight L Brandl T Huber B Henrich G. Psychological problems of cancer patients: a cancer distress screening with a cancer-specific questionnaire. Br J Cancer. (2004) 91(3):504–11. doi: 10.1038/sj.bjc.6601986 PMC2409853 15238979

[53] Acquati C Kayser K. Predictors of psychological distress among cancer patients receiving care at a safety-net institution: the role of younger age and psychosocial problems. Support Care Cancer (2017) 25(7):2305–12. doi: 10.1007/s00520-017-3641-8 28255807

[54] Kim SJ Rha SY Song SK Namkoong K Chung HC Yoon SH. Prevalence and associated factors of psychological distress among Korean cancer patients. Gen Hosp Psychiatry (2011) 33(3):246–52. doi: 10.1016/j.genhosppsych.2011.02.008 21601721

[55] Chiou YJ Chiu NM Wang LJ Li SH Lee CY Wu MK. Prevalence and related factors of psychological distress among cancer inpatients using routine distress thermometer and Chinese health questionnaire screening. Neuropsychiatr Dis Treat (2016) 12:2765–73. doi: 10.2147/NDT.S118667 PMC5087777 27822049

[56] Ise M Nakata E Katayama Y Hamada M Kunisada T Fujiwara T. Prevalence of psychological distress and its risk factors in patients with primary bone and soft tissue tumors. Healthcare (Basel) (2021) 9(5):566. doi: 10.3390/healthcare9050566 34065006 PMC8151264

[57] Lenze F Kirchhoff C Pohlig F Knebel C Rechl H Marten-Mittag B. Standardized screening and psycho-oncological treatment of orthopedic cancer patients. In Vivo (2018) 32(5):1161–7. doi: 10.21873/invivo.11359 PMC6199587 30150439

[58] International Agency for Research on Cancer. Gaza Strip and West bank facts sheet 2021 (2021). Available at: https://gco.iarc.fr/today/data/factsheets/populations/275-gaza-strip-and-west-bank-fact-sheets.pdf.

[59] Abu-Odah H Molassiotis A Liu JYW. Assessment of the educational and health care system–related issues from physicians' and nurses' perspectives before developing a palliative care program within the Palestinian health care system: A cross-sectional study. J Hospice Palliative Nurs (2022) 24(3):E59–E75. doi: 10.1097/NJH.0000000000000840 35085161

[60] Abu-Odah H Molassiotis A Yat Wa Liu J. Analysis of the unmet needs of Palestinian advanced cancer patients and their relationship to emotional distress: results from a cross-sectional study. BMC Palliative Care (2022) 21(1):72. doi: 10.1186/s12904-022-00959-8 35562732 PMC9106510

[61] Abu-Odah H Molassiotis A Zhao IY Su JJ Allsop MJ. Psychological distress and associated factors among Palestinian advanced cancer patients: A cross-sectional study. Front Psychol (2022) 13:1061327. doi: 10.3389/fpsyg.2022.1061327 36533049 PMC9755485

[62] National Institute for Health and Care Excellence. Recommendations organised by symptom and findings of primary care investigations. suspected cancer: recognition and referral (2021). Available at: https://www.nice.org.uk/guidance/ng12/chapter/Recommendations-organised-by-symptom-and-findings-of-primary-care-investigations#respiratory-symptoms.

[63] Igarashi H Fukushi M Nago N. Oxygen use and survival in patients with advanced cancer and low oxygen saturation in home care: a preliminary retrospective cohort study. BMC Palliative Care (2020) 19(1):3. doi: 10.1186/s12904-019-0511-9 31900147 PMC6942361

[64] Hayama M Suzuki H Shiroyama T Tamiya M Okamoto N Tanaka A. Chemotherapy for patients with advanced lung cancer receiving long-term oxygen therapy. J Thorac Dis (2016) 8(1):116–23. doi: 10.3978%2Fj.issn.2072-1439.2016.01.42 10.3978/j.issn.2072-1439.2016.01.42 PMC4740147 26904219

[65] Cullen DL Stiffler D. Long-term oxygen therapy: review from the patients' perspective. Chron Respir Dis (2009) 6(3):141–7. doi: 10.1177/1479972309103046 19643828

